# Novel axolotl cardiac function analysis method using magnetic resonance imaging

**DOI:** 10.1371/journal.pone.0183446

**Published:** 2017-08-24

**Authors:** Pedro Gomes Sanches, Roel C. op ‘t Veld, Wolter de Graaf, Gustav J. Strijkers, Holger Grüll

**Affiliations:** 1 Biomedical NMR group, Department of Biomedical engineering, Eindhoven University of Technology, Eindhoven, the Netherlands; 2 Department of Biomaterials, Radboud University Medical Center, Nijmegen, the Netherlands; 3 Department of Biomedical Engineering and Physics, Academic Medical Center, University of Amsterdam, Amsterdam, the Netherlands; 4 Department of Radiology, University Hospital of Cologne, Cologne, Germany; Semmelweis Egyetem, HUNGARY

## Abstract

The salamander axolotl is capable of complete regeneration of amputated heart tissue. However, non-invasive imaging tools for assessing its cardiac function were so far not employed. In this study, cardiac magnetic resonance imaging is introduced as a non-invasive technique to image heart function of axolotls. Three axolotls were imaged with magnetic resonance imaging using a retrospectively gated Fast Low Angle Shot cine sequence. Within one scanning session the axolotl heart was imaged three times in all planes, consecutively. Heart rate, ejection fraction, stroke volume and cardiac output were calculated using three techniques: (1) combined long-axis, (2) short-axis series, and (3) ultrasound (control for heart rate only). All values are presented as mean ± standard deviation. Heart rate (beats per minute) among different animals was 32.2±6.0 (long axis), 30.4±5.5 (short axis) and 32.7±4.9 (ultrasound) and statistically similar regardless of the imaging method (p > 0.05). Ejection fraction (%) was 59.6±10.8 (long axis) and 48.1±11.3 (short axis) and it differed significantly (p = 0.019). Stroke volume (μl/beat) was 133.7±33.7 (long axis) and 93.2±31.2 (short axis), also differed significantly (p = 0.015). Calculations were consistent among the animals and over three repeated measurements. The heart rate varied depending on depth of anaesthesia. We described a new method for defining and imaging the anatomical planes of the axolotl heart and propose one of our techniques (long axis analysis) may prove useful in defining cardiac function in regenerating axolotl hearts.

## Introduction

In humans and most other mammals, the regenerative potential is limited and often restricted to certain tissues. Examples for cyclical regeneration include maintenance of skin, renewal of the intestinal lining, new blood vessel formation, hair, bone, as well as neurogenesis in the brain [1–5]. However, many diseases and accidents may result in damaged tissues, organs, limbs, or other crucial structures that do not (fully) regenerate or restore their original functional capacity. This is the case in cardiac remodelling following heart injury [6]. Dead cardiomyocytes are not regenerated but instead replaced by scar tissue which does not provide the necessary electro-conductivity and contractile properties of the healthy beating heart. The American Heart Association reports that cardiovascular disease is responsible for 30.8% of deaths in the US, and that its risk factors like smoking and obesity are at an all-time high [7]. Current treatments include the placement of new heart valves to replace damaged ones or implanting a pacemaker to correct an arrhythmia [8,9]. Currently, there is no viable treatment for heart failure other than heart transplant. These treatments have in common that they are highly invasive and replace, rather than regenerate, lost functions.

**Fig 1:**
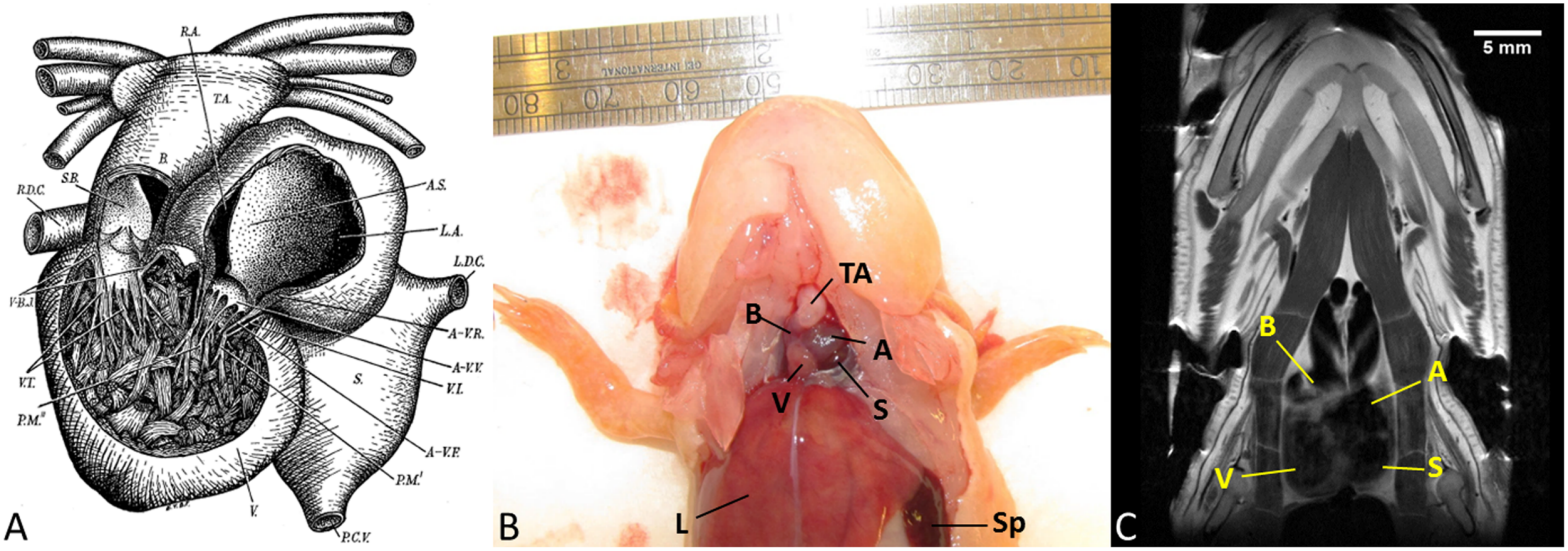
Anatomy of the axolotl heart. (A) Anatomical drawing [16], the sinus (*S*.) sends collected bodily blood to the left atrium (*L*.*A*.) while the right atrium (*R*.*A*.) receives oxygenated blood from the lungs and gills. Both atria simultaneously pump the blood into the ventricle (*V*.), where a dense network of muscle fibres will contract to squeeze out the blood into the bulbus cordis (*B*.) and then truncus arteriosus (*T*.*A*.). (B) Euthanized axolotl in ventral view. The heart is located close to the throat, more cranially than the heart of most mammals. (C) Coronal section of an axolotl scanned with a T2 weighted MRI sequence. The three images aid in understanding the anatomy of the axolotl heart. A = atria, V = ventricle, S = sinus, B = bulbus cordis, TA = truncus arteriosus, L = liver, Sp = spleen. Panel (A) was reproduced and reprinted with permission from The Royal Society of London from reference [16].

Fortunately, not all vertebrates suffer from this limited regenerative potential. The axolotl (*Ambystoma mexicanum*), an aquatic salamander, is known for being able to fully regenerate many body parts, including the limbs [10], tail [11], spinal cord [12] and even organs such as the heart [13]. A recent study showed cardiac regeneration of the axolotl heart relies on similar mechanisms as the regeneration of other body parts; namely on the dedifferentiation and then proliferation of resident cells [14]. They also highlight that epigenetic regulation of Baf60c is of importance in the scar-free healing of both axolotl and neonatal mouse hearts. Indeed, it has been proposed that many factors, such as nerves, amount of resident fibroblasts, or the hippo pathway, are responsible for the distinguished regeneration mechanisms of the axolotl [15]. As an amphibian, the axolotl’s heart is characterized by one ventricular cavity, an atrium separated by a partly perforated septum, and the absence of coronary circulation (Fig 1) [16]. The left atrium contracts slightly earlier than the right, causing its oxygen-poor blood to be positioned more closely to the exit of the ventricle. When ventricular contraction occurs, this oxygen-poor blood is first to leave the heart. It will then primarily enter the pulmonary veins due to the low wall pressure of these veins at the start of contraction. This way, oxygen-poor blood can be resupplied with oxygen in the gills and the lungs while the oxygen-rich blood is sent to the rest of the body. The amphibian heart functions in a similar manner to the mammalian one but without a ventricular septum. One striking difference, however, is that most contractile power in mammals comes from the myocardium whereas amphibians rely on the contraction of a complicated network of muscle fibres inside the ventricle.

The regenerative capacity of the axolotl’s heart was elegantly demonstrated by Cano-Martinez *et al*. in 2010. The ventricle was partially amputated and then allowed to regenerate. The heart was then excised, placed in an isolation organ chamber and the cardiac function was measured *ex vivo* with a polygraph. A near complete return of heart morphology and contractile activity was observed and this highlighted the potential for the axolotl as a model organism for studying heart regeneration mechanisms. Unfortunately, these data were acquired *post-mortem* and the method thus did not facilitate tracking of the regeneration process over time within an animal. An ideal approach would use non-invasive imaging techniques that allow longitudinal studies where regeneration may be monitored *in vivo* without the need to sacrifice animals in each time point. For example, fluorescent microscopy techniques have been used in the past to monitor regeneration of the spinal cord in translucent GFP-expressing axolotls [17]. Magnetic resonance imaging (MRI) was previously employed by Kropf and colleagues on axolotls with sciatic nerve injury [18]. However, thereafter the use of MRI imaging in axolotls has been surprisingly absent.

Therefore, we have investigated the potential of MRI to study the axolotl’s heart function. Heart function is often assessed by measuring ejection fraction (EF), or the heart rate (HR) and stroke volume (SV) which may be multiplied to obtain cardiac output (CO). To this end we used a retrospectively gated T1-weighted sequence, commonly used for determining HR, EF, and CO in small animal models such as mice [19]. We present methods for handling juvenile axolotls during multi-hour imaging scans, defining the heart axes (needed for heart function quantification) and calculating heart function based on MRI data.

## Materials & methods

All reagents and solvents were obtained from Sigma-Aldrich^®^ (Steinheim, Germany)) and used without further purification, unless stated otherwise. Holtfreter’s solution (100%) was prepared as follows: 3.46 g NaCl, 0.05 g KCl, 0.1 g CaCl_2_ and 0.2 g MgSO_4_·7H_2_O in 1 L MilliQ^®^ water and the pH adjusted to 7–8 using NaHCO_3_. Dilutions for aquarium water were made with tap water (pre-treated with sera^®^ Toxivec) and for all other uses with MilliQ^®^ water.

### Animals

Male and female golden albino axolotls (*Ambystoma mexicanum*) were obtained from the Ambystoma Stock Centre (Lexington, KY). The animals were individually housed in 40% Holtfreter’s solution at 18°C in our animal facility (S1 Fig). At the time of scan, all animals were 16 months old and weighed 81.1 ± 1.9 g (mean ± S.D.).

This study, including all animal procedures, was approved by the ethical review committee of the Maastricht University Hospital, the Netherlands, (approval number: DEC 2012–058) and was performed according to the principles of laboratory animal care (Institute of Laboratory Animal Resources (U.S.) Committee of Care and Use of Laboratory Animals) and the Dutch national law “Wet op Dierproeven” (Stb 1985, 336).

#### Animal preparation

Animals were anaesthetized by immersion in 0.03% benzocaine solution (dissolved from powder in 40% Holtfreter’s solution containing 1% ethanol) until absence of the righting reflex. The anaesthesia was then maintained by wrapping the animal in 0.015% benzocaine-soaked Kim-wipes^™^ (Kimberly-Clark Professional^®^, Roswell, Canada). The wrapped animals were placed inside a Minerve^®^ (Esternay, France) scan bed. Given the lack of external visual cues regarding the well-being of the animals under anaesthesia, the heart rate of the animals was checked with a CL15-7 ultrasound probe (HDI5000, Philips, Bothell, WA). The probe was placed just below the animal’s head to visualize the contracting ventricle. The number of contractions in one minute was counted by eye to *calculate the HR*. No other measurements were made with the ultrasound device. Animals were sacrificed by intraperitoneal injection of sodium pentobarbital (200 mg/kg body weight).

### MRI scanning protocols

A 9.4 T Bruker Biospec^®^ (Ettlingen, Germany) small-animal MRI scanner with Paravision^®^ (v5.1) software was used with a 1H 35-mm-diameter Quadtransceiver radiofrequency coil (Bruker Biospin^®^) for imaging. A fast T_1_-weighted RARE (Rapid Acquisition with Relaxation Enhancement) survey scan was utilized to start slice planning of the cardiac motion scans (repetition time = 2005 ms, echo time = 8.5 ms, number of averages = 1, field of view = 4.6*3 cm^2^, matrix = 256*256, voxel size = 180*117 μm^2^, slice thickness 0.5 mm). A retrospectively-triggered FLASH (Fast Low Angle Shot) cine sequence [20] was used to obtain 25-frame cardiac motion sequences (sequence = intragate-FLASH, repetition time = 6 ms, echo time = 2.9 ms, field of view 3*3 cm^2^, matrix = 256*256, voxel size = 117*117 μm^2^, slice thickness = 1 mm, number of repetitions = 250, total acquisition time = 4 min). No respiratory gating was required since respiratory motions are absent in anaesthetized axolotls. Anatomical images were obtained with a T_2_-weighted Turbo-RARE sequence (repetition time = 5430 ms, echo time 36 ms, number of averages = 4, field of view = 4.5*3 cm^2^, matrix = 384*384, voxel size = 117*78 μm^2^, slice thickness = 0.5 mm).

#### Heart axes acquisition

**Fig 2:**
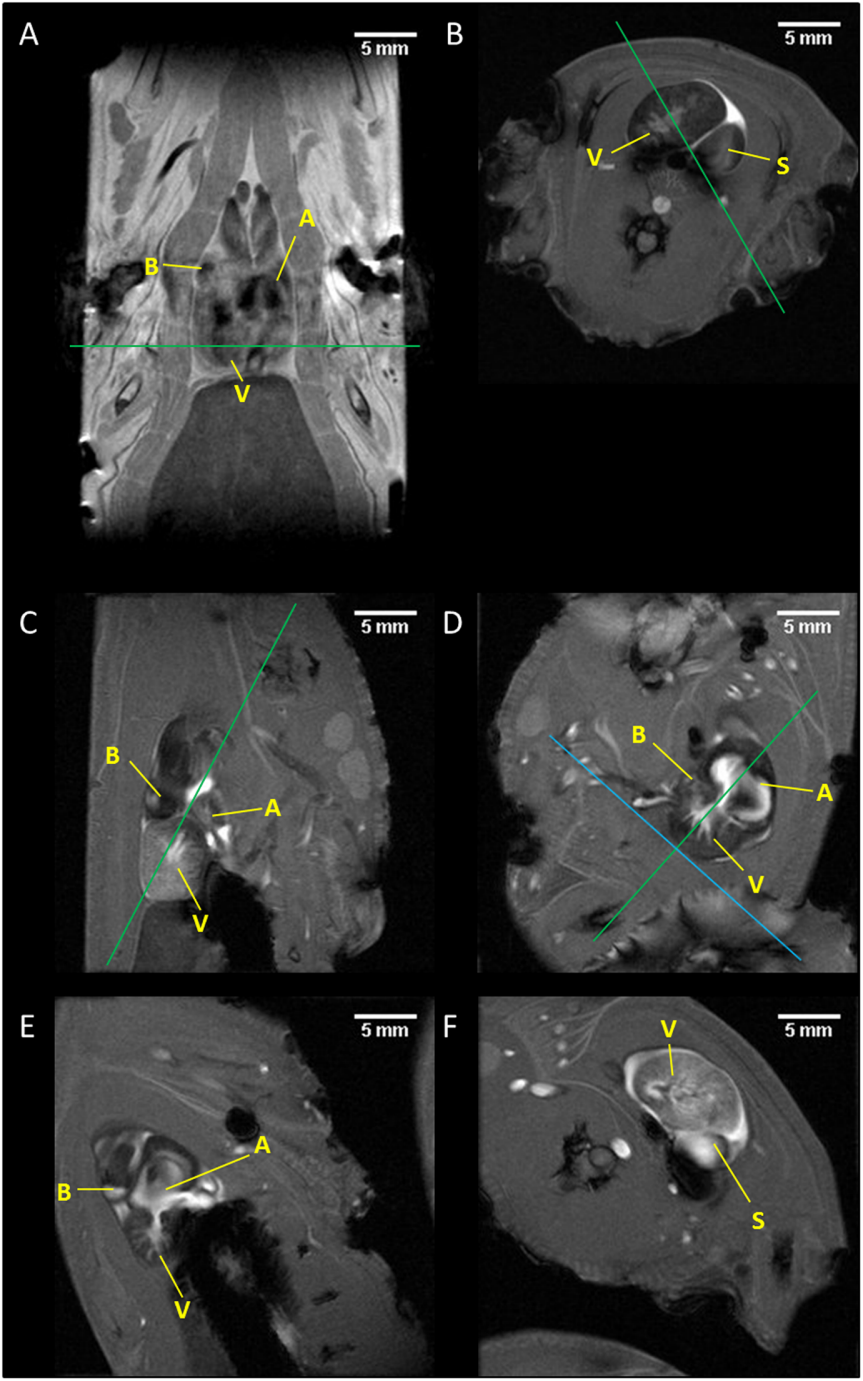
A step-by-step procedure to obtain all axes of the axolotl heart. (A) A pilot scan (T_1_-weighted) was used to locate the heart. (B) An axial FLASH scan is positioned around the middle or lower end of the ventricle—green line in (A); a scan perpendicular to (B) is positioned to cross the ventricular wall in the ventral side and the dorsal intersection of the ventricle and sinus. This yields the apparent long axis (C). The next scan is made perpendicular to this, intersects the apex of the heart and then crosses through the three-way intersection of the ventricle, atria and bulbus to yield the vertical long axis (D). A scan perpendicular to this that crosses the AV valve and the apex of the ventricle is considered the horizontal long axis (E). The short axis was then be obtained by planning a series of scans covering the whole heart that are at a 90° degree angle from the horizontal long axis (panel F, the 7^th^ slice in a short axis series, displays the ventricle). Green lines represent the slice orientation for the next scan. In panel D, the green line shows the orientation for the horizontal long axis of panel E and the blue line shows the first slice of the short axis scan series of panel F. A = atria, V = ventricle, S = sinus, B = bulbus cordis.

For three axolotls, long and short axes of the axolotl’s heart were defined that were equivalent to the mammalian longitudinal and short axes, respectively (Fig 2) [21]. It was along these axes that scans were performed to extract the functional cardiac information. Each animal was scanned three times within one anaesthesia moment, and the axes were defined for each scan. After every scan, the animal was briefly removed from the scanner to measure its heart rate with US. When placing the animal bed back in the MRI, it was tilted intentionally with respect to the previous position. This was done in order to ensure that our methods are robust, and can successfully result in the acquisition of the heart axes regardless of orientation of the animal.

First, an axial FLASH scan was performed on the caudal side of the heart that intersected both the ventricle (V) and sinus (S). Next, the apparent long axis was obtained by planning a slice on the previous scan to pass through the dorsal intersection of the walls of the ventricle and sinus. The second intersection of this slice was placed approx. half-way through the opposite side of the ventricle wall during diastole. The vertical long axis was then obtained by crossing a slice through the apex and intersection of the atria (A), ventricle and bulbus cordis of the previous scan. The horizontal long axis was then defined as perpendicular to the previous scan, and was rotated to pass through the atrial-ventricular valve. The short axis of the heart was a 90° rotation from the horizontal long axis and was repeated in a series of scans until the whole heart was covered.

### Data analysis

To determine the cardiac function we calculated three quantities: Stroke Volume (*SV* = *EDV* − *ESV*), Ejection fraction $\left(EF\;=\frac{SV}{EDV}\;*100\%\right)$, and Cardiac Output (*CO* = *SV* * *HR*). Where SV is stroke volume in μL/beat, EF is ejection fraction in %, EDV is End Diastolic Volume in mL/beat, ESV is End Systolic Volume in mL/beat, and CO is cardiac output in mL/min. Each quantity was calculated via two different methods based on the heart axes defined above: Method 1 –combined long axis (LA) which combines data from two scans, the vertical and the horizontal long axes (VLA and HLA). Method 2 –short axis series (SA) which combines a series of scans of the ventricle scanned cranial-to-caudal along the short axis direction. Heart rates (HR) were determined using Paravision^®^’s built-in Intragate tool. Cardiac function was quantified by semi-automatic analysis using QMassMR^®^ v7.6 software (Mediso medical imaging systems Ltd., Leiden, The Netherlands). Heart rates were also visually measured with ultrasound, as described previously, immediately before and after placing the animal in the MRI scanner.

The contours of the inner ventricular muscle mass, individually drawn for each image slice, served as a base for cardiac function analysis. Based on the dynamic MRI videos of the beating heart the mostly static outer muscle volume was kept outside the delineated volumes. At end diastole (ED) and end systole (ES) the delineation was made including the whole contractile volume, since the blood permeates the trabecular cardiac muscle. This delineation process was applied for the VLA, HLA, and SA orientations. For the latter, slices that did not contain portions of the ventricle during either ES or ED were excluded from their respective analysis. The calculated ED/ES volumes were used to estimate EF, SV, and HR was also used for CO.

### Statistical analysis

Our data were paired as we performed measurements over two directions (long and short axis) for every scan, which resembles a randomized block design [22]. The non-parametric Kruskal-Wallis test was used to test for significant differences in HR, SV, and EF attributed to animal physiology and experimental repeat. In our design, experimental repeat both indicates whether our acquisition method was consistent and if there was an effect of anaesthesia time. The Wilcoxon signed-rank test was performed to test for differences between the methods (long and short axis, and ultrasound). Cardiac output was omitted from analysis, as it is calculated using SV and HR and any statistical significant changes might be the result of fluctuating HR, rather than differences in acquisition and/or analysis methods. All analyses were made using SPSS (IBM Corporation, New York, NY), using a significance level α of 0.05. Values are presented as mean ± S.D.

## Results

All three axolotls were subjected to MRI scanning sessions of 4.5 hours. The heart rate immediately after inducing anaesthesia was approx. 50 bpm. After the first hour the HR would drop to about 30 bpm and typically drift around this value (± 5) for the remainder of the scanning session. EF and SV are based on volumetric differences in the ventricle before and after contraction and appear unaffected by fluctuations in heart rate. On the other hand, CO is based on HR and SV. We therefore cannot draw any conclusions from CO values, due to HR variability under anaesthesia. The values of the different cardiac function parameters are summarized in Table 1.

**Table 1:** The cardiac function parameters of the three axolotls, as measured by MRI and US. For US, the heart rate was calculated before and after the MRI scans.

Animal	Repeat	Cardiac function parameter	MRI		Ultrasound (before-after MRI scan)
			(Combined) Long axis	Short axis	
1	1	HR	40.0	41.0	42–42
		SV	110.1	95.6	-
		EF	58.0	50.8	-
		CO	4.4	3.9	-
	2	HR	36.0	26.0	30–30
		SV	154.5	136.2	-
		EF	67.1	57.9	-
		CO	5.6	3.5	-
	3	HR	24.5	25.0	27–27
		SV	84.2	61.9	-
		EF	49.9	32.0	-
		CO	2.1	1.6	-
2	1	HR	40.5	35.0	42–31.5
		SV	80.5	79.8	-
		EF	48.1	47.0	-
		CO	3.3	2.8	-
	2	HR	31.0	34.0	31.5–27
		SV	159.8	59.7	-
		EF	62.8	35.2	-
		CO	5.0	2.0	-
	3	HR	29.0	24.0	27–31,5
		SV	144.6	67.9	-
		EF	58.3	38.3	-
		CO	4.2	1.6	-
3	1	HR	37.5	31.5	45–37,5
		SV	145.3	145.2	-
		EF	63.7	66.1	-
		CO	5.5	4.6	-
	2	HR	27.0	28.5	30–30
		SV	173.7	108.0	-
		EF	66.3	56.8	-
		CO	4.7	3.1	-
	3	HR	28.0	29.0	30–30
		SV	150.8	84.7	-
		EF	62.8	48.5	-
		CO	4.2	2.5	-

HR = heart rate (bpm), SV = Stroke Volume (μL/beat), EF = ejection fraction (%), CO = cardiac output (ml blood / min).

**Fig 3:**
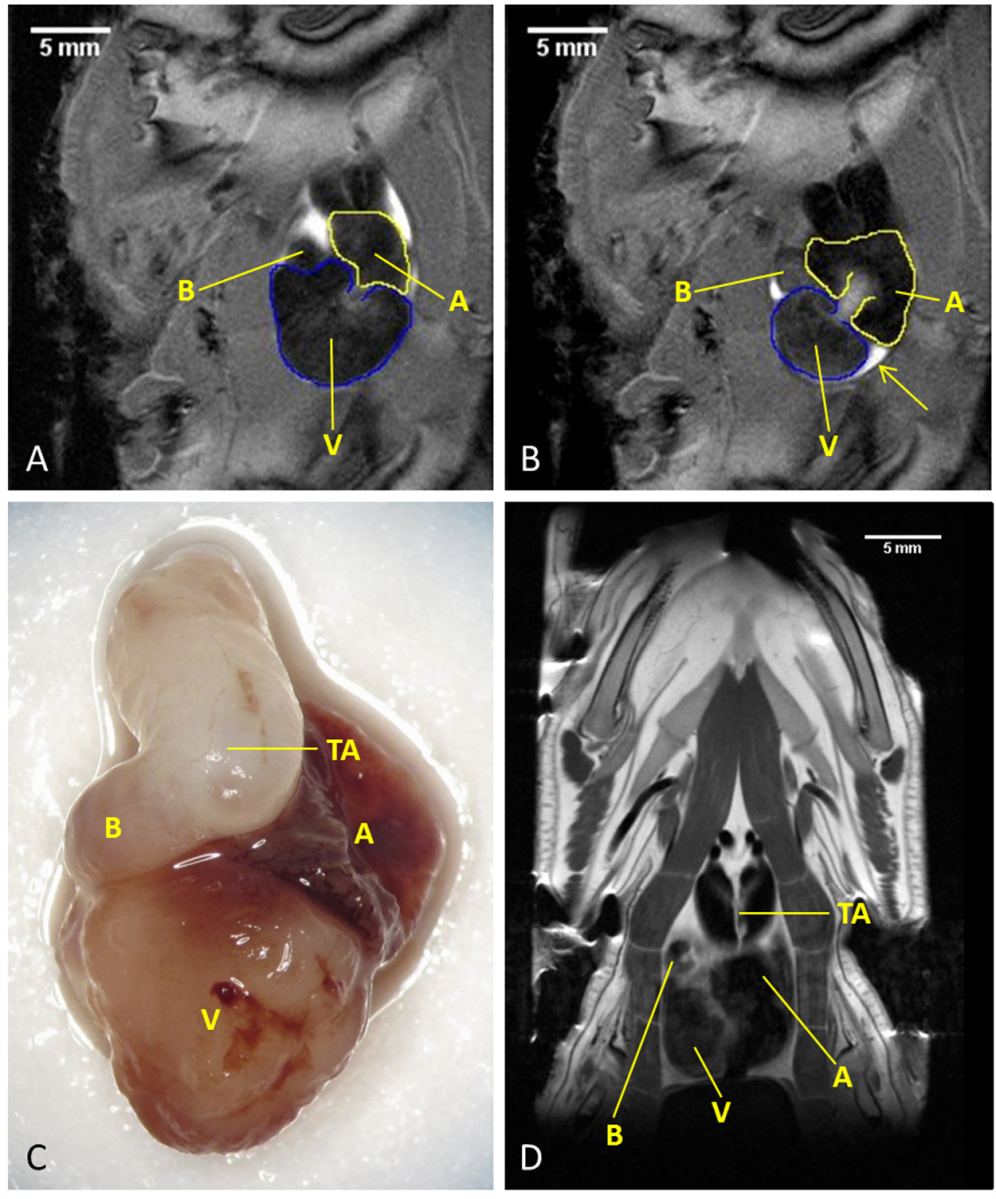
**Vertical long axis of the axolotl heart at ED (A) and ES (B)**. During the cardiac cycle the atria (yellow contours) and inner ventricular muscle mass (blue contours) expand and fill with blood sequentially, whereas the outer cardiac walls remain relatively stationary. *Ex vivo* photograph of an excised axolotl heart (C) and T2 weighted anatomical MR image of the heart in the coronal plane (D). The orientation of the ventricle to the atria and bulbus cordis differs between the MRI and photograph because the heart is oriented differently in the animal. A = atria, V = ventricle, B = Bulbus Cordis, TA = Truncus Arteriosus.

**Fig 4:**
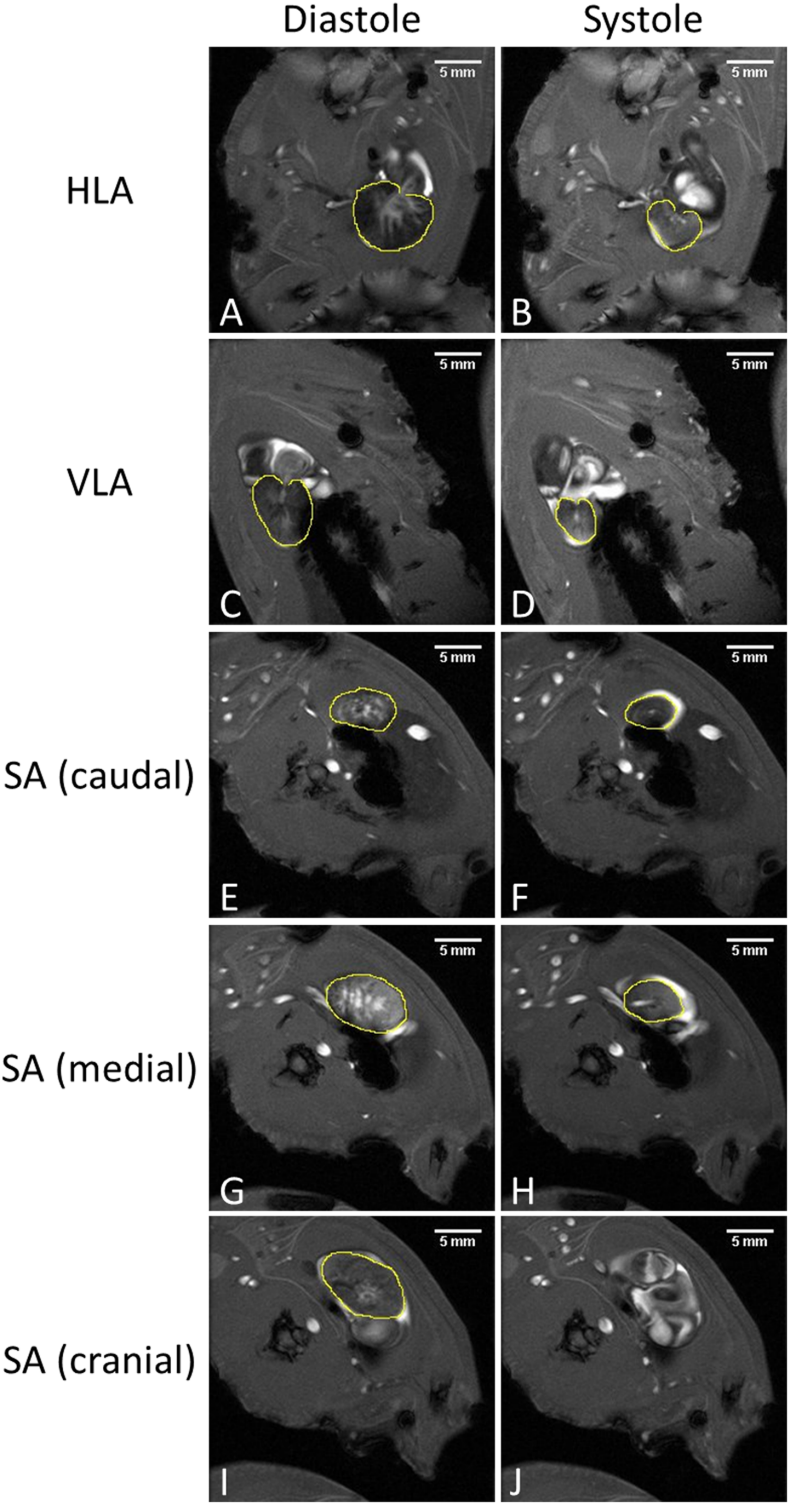
Examples of contours drawn around the inner ventricular muscle mass during ED and ES for cardiac function analysis. Note how this muscle network expands and shrinks, while the outer cardiac walls stay relatively stationary (as indicated by the yellow contours). Horizontal long axis (A & B), Vertical long axis (C & D), a slice close to the apex of the heart (E & F), a slice at the centre of the ventricle (G & H) and a slice at the tip of the ventricle (I & J). No contour is drawn in panel J, because the ventricle is moved out of the slice and it now shows the atria. The slice shown in J was excluded for analysis in the ES but still included for ED.

Representative MR images of an axolotl’s heart at ES and ED (along the coronal axis) are shown in Fig 3 as well as an *ex vivo* photograph and a T2 weighted coronal slice through the heart. To calculate the cardiac function both ES and ED frames were identified and the ventricle delineated for every orientation axis. Examples of these delineations can be seen in Fig 4. A full 25-frame cardiac cycle is shown in S2 Fig.

**Fig 5:**
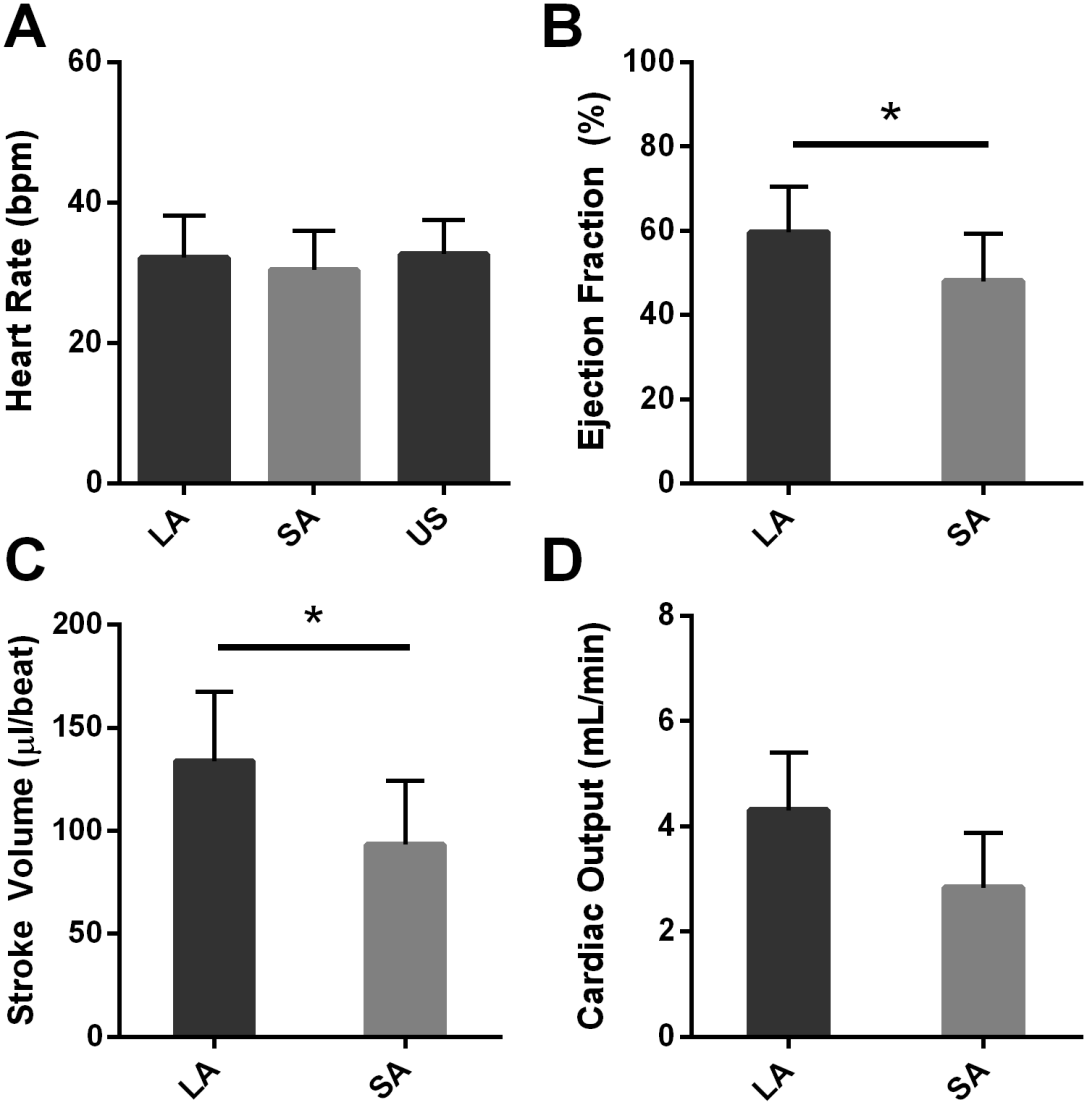
Average HR (A), EF (B), SV (C), and CO (D) calculated by LA, SA or US techniques. Wilcoxon signed-rank tests showed significant differences between LA and SA methods for both EF and SV (p 0.015 and 0.008) but not between LA, SA, and US methods for HR (p = 0.374, 0.779, and 0.066 for LA vs SA, LA vs US, and SA vs US).

The cardiac function values (HR, EF, SV, and CO) for the different acquisition techniques (LA, SA, & US) are summarized in Fig 5. The numerical values can also be found in S1 Table. The HR measurement did not differ between MRI and US methods. However, calculated EF and SV were different between the LA and SA methods (p > 0.05).

In S2 Table the results of the Kruskal-Wallis and Wilcoxon tests for the influence of animal, experimental repeat, and scanning technique are listed. EF, SV, and HR did not differ between animals. The experimental repeat only affected the results for HR.

## Discussion

We have shown the implementation of a cardiac function analysis method using MRI in axolotls. To this end, a retrospectively triggered gated FLASH sequence, which had already been established in several mouse studies, was optimized [20,23,24]. MRI scanning parameters were similar to those used in mice because of the comparable body sizes of axolotls and mice. An important difference, however, is that the axolotl has a resting heart rate of typically 50 bpm, while that of a mouse may vary between 450 and 500 bpm [16,25]. In addition, respiratory motion in the anaesthetized axolotl was absent which made respiratory gating unnecessary. Cardiac slice planning and data analysis proved to be challenging for two reasons. First, the heart of salamanders only possesses one ventricle and is shaped differently from rodents. Thus, the separations between cardiac chambers had to be investigated and new definitions for the heart’s principal axes had to be made. Second, the contractile function of a salamander ventricle originates almost exclusively from the muscular network found inside the lumen, rather than the ventricular walls [16]. Contraction of this inner ventricular muscle squeezes the blood out into the *bulbus cordis*, where distribution of oxygenated and de-oxygenated blood into the body and pulmonary arteries relies on spatial and temporal distribution from pressure differences [16]. In a similar manner, the walls of the sinus and atria consist of a basket-weave of muscle fibres providing most of the contractile work. This became evident when visualizing the contraction with MRI. The outer cardiac walls remain relatively stationary while the inner structures (ventricle, atria) enlarge and shrink in sequence. Contractile motion videos, as captured by MRI, are available in the online version of this article (S1–S3 Videos). We found that the step-wise manner of slice planning (used to obtain the HLA, VLA, and SA (Fig 2)) could be repeated consistently and provided highly comparable results each time.

In order to analyse cardiac MRI data of axolotls, we discovered standard methods and definitions used for mice could not be used in axolotls due to the anatomical and functional differences between the axolotl and mouse heart. Firstly, due to the lack of contractile motions from the ventricular wall, drawing contours around the ventricular wall was not a feasible approach to calculate volume changes during contraction. Visually inspecting the cardiac contraction movies revealed that the inner ventricular muscle mass almost exclusively shows motion, while the ventricular wall stays relatively stationary. Therefore, a new method was developed for calculating volume changes of the inner ventricular muscle mass. Secondly, in some SA images the separation between ventricle and atria was not clearly distinguishable. Scanning with higher spatial resolution would probably show the separation more clearly. However, in our study this would have resulted in longer scans that would no longer allow us to perform three separate scans within one anaesthesia session (max. 5h). Thirdly, it proved difficult to distinguish between ED and ES in the SA slices closest to the apex of the heart. This resulted in some exclusion of heart tissue that contributes to contraction, ultimately under-estimating the values of our cardiac function parameters with the SA method. Our suggestion would be that, given this software package, one should avoid using the SA method since it is prone to underestimate the contraction volumes. Indeed, when testing the reliability and comparability of our two analysis technique (LA and SA), we found that there was a significant difference. The SA method consistently resulted in lower values than the LA method in all but one case. For EF, this variation was within 7 to 28% (absolute value), while for SV this was within 14 to 101 μl. In some cases, this was a difference of up to factor 2. We believe this is the result of the aforementioned problems associated with the SA method. These may lead to omitting functional parts of the ventricle and thus to an underestimation of cardiac function. Fortunately, there is no indication that any of these issues affected the combined long axis method.

All three measurement techniques for the HR (US and cardiac MRI in long and short axis orientation) returned comparable results. The three animals, which were of the same age and had comparable body weight, possessed similar HRs in all three experimental repeats. However, there was a statistical difference between the first experimental repeat when compared to the second and third. This may be explained by the fact that the first experimental repeat was obtained shortly after induction of anaesthesia. The second and third repeats were typically performed approx. 2 and 3 hours after induction of anaesthesia, respectively. We observed in numerous axolotl experiments (not reported here) that when ample anaesthetic solution was provided, the HR would drop from the expected 50 bpm at rest to 30 bpm. Still, it was not uncommon for the axolotl to still have a HR closer to 50 bpm at the start of an anaesthesia period, even when the righting reflex was already absent. Most likely, the element of time that the experimental repeat implies is representing three levels of anaesthetic depth: weak, medium and strong. Our data seem to suggest that the timing of a cardiac function measurement after anaesthesia induction is relevant when comparing the results of scans. For future studies, we advise to keep the depth of anaesthesia as consistent as possible among experiments and follow a tight time schedule, to minimize HR variability (S3 Fig). Regardless, no significant differences were found for SV and EF in relation to the repeat number or between axolotls. These results also show that our methodology to determine the heart axes was robust as it was possible to define them by following our methodology, regardless of the orientation of the axolotl in the scanner (S4 Fig).

Nonetheless, some limitations to this study must be pointed out: (1) the axolotls were scanned at a certain point and as such age/weight dependent heart function values have not been determined; (2) an *ex vivo* reference test was not performed to compare the MRI-based calculated values. In fact, there is a lack of studies reporting heart function values in juvenile/adult axolotls [26,27]. For comparison, US Doppler measurements performed on un-anaesthetized *Ambystoma tigrinum* weighing an average 105 g (ours weighed approx. 81 g) returned mean stroke volume (0.18 ml ± 0.06) and cardiac output (11.0 ml ·min^−1^ ± 3.6) [28]. The limited studies that exist show that for *Xenopus* the SV and CO increase with increasing body mass during most of development [27]. A similar relationship can be expected for other amphibians, such as axolotls, which would help justify the higher values of SV and CO found in the more massive *Ambystoma tigrinum*. Even though the data seem to suggest that our *in vivo* MRI-based values are close to the expected physiological parameters we do not know the accuracy of our values. Further research is needed in order to assess the reliability of using the calculated cardiac function parameters as absolute physiological values; (3) the number of animals used should be increased in future studies to strengthen the statistical analysis.

We consider the MRI-based methodology employed here to be robust and therefore suggest that our definition of the vertical and horizontal long axes can be combined for cardiac function measurements in axolotls. This method could be useful for assessing the state of cardiac regeneration, as one can compare e.g. the EF and SV before-and-after inflicting cardiac injury. Since it has been shown that cardiac contractile activity recovery positively correlates with cardiac regeneration *ex vivo*, we expect to see a similar correlation using cardiac MRI [13]. If fluctuations in HR are minimized, CO may also prove an interesting factor to investigate.

## Conclusion

We have designed and implemented a methodology to non-invasively quantify cardiac function in axolotls. This method can be employed to study heart recovery from injury in longitudinal studies performed on the same animal. Studies such as these may contribute to better understanding of cardiac regeneration methods employed by these animals, and the possible application of such methods in the treatment of human cardiac injury.

## Supporting information

S1 Fig Axolotl aquarium setup of the Eindhoven University of Technology, Department of Biomedical Engineering, Biomedical NMR group. Up to 15 axolotls could be individually housed with flowing water in this setup. All cages were cleaned daily to remove faeces, leftover food and dust particles.
(TIF)

S2 Fig Example of a 25 frame cardiac movie in the horizontal long axis plane of an axolotl heart. The heart is in systole in frame 1 and reaches diastole in frame 10. The order in which the atria, then ventricle and lastly the bulbus cordis & truncus arteriosus swell up with blood is very prominent when watching the frames as a short film (see S1 Video).
(TIF)

S3 Fig HR, SV and EF, measured by long axis method, plotted out for each repeat. (TIF)

S4 Fig A comparison between systole and diastole images of both HLA and VLA of one animal. This image illustrates the consistency among repeated scans of the same animal. Mainly the size and position of the heart compartments are of importance when calculating the various cardiac function parameters.
(TIF)

S1 Table Average value of HR, EF, SV and CO for each analysis technique. (DOCX)

S2 Table Kruskal-Wallis and Wilcoxon tests for significant differences attributed to animal physiology, experimental repeat, and scanning technique. None of the cardiac function factors appeared to differ between the different animals and experimental repeats. This excludes experimental repeat of HR, in which the first experimental repeat appears to differ from the second and third. Using the LA or SA analysis techniques significantly affects the measurement of SV and EF, whereas HR was similar for LA, SA and US techniques. Bold = significant.
(DOCX)

S1 Video Cardiac motion video captured using the MRI cine sequence. This image was captured slightly off-plane from a coronal section of the body. The blood can be seen traveling from the atria to the single ventricle, before exiting the heart through the bulbus cordis. Motions seen at either the bottom or top of the screen result are the result of imaging artefacts.
(GIF)

S2 Video Cardiac motion video captured using the MRI cine sequence. This image is angled further from the coronal section of the body, and approaches the sagittal plane. The ventricle lies more predominantly in view, and the inner basket-weave structure of this compartment is highlighted. Motions seen at either the bottom or top of the screen are the result of imaging artefacts.
(GIF)

S3 Video Cardiac motion video captured using the MRI cine sequence. This sagittal view reveals three of the compartments of the heart, namely the ventricle (lower left), one of the atria (top), and the sinus (lower right). Note how the atria fill up and blood is pushed into the ventricle. Simultaneously with the ventricle, the sinus will receive blood (coming back from the rest of the body). Motions seen at either the bottom or top of the screen are the result of imaging artefacts.
(GIF)
