# Supplementary figures and images for: Novel axolotl cardiac function analysis method using magnetic resonance imaging
Source: PLoS One. 2017 Aug 24;12(8):e0183446. doi: 10.1371/journal.pone.0183446 (PMC5570274; doi:10.1371/journal.pone.0183446)

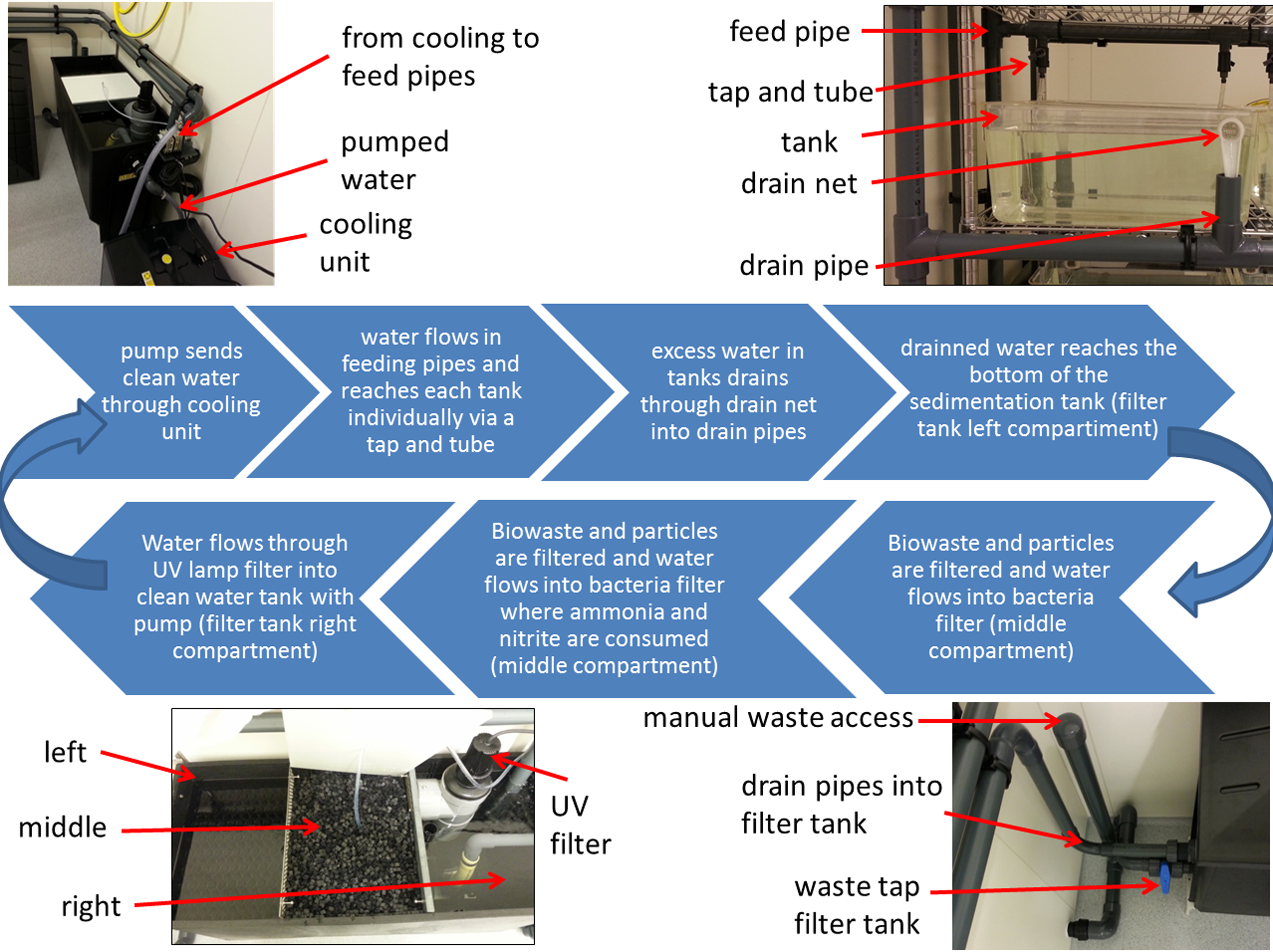

Supplement: S1 Fig — Up to 15 axolotls could be individually housed with flowing water in this setup. All cages were cleaned daily to remove faeces, leftover food and dust particles. (TIF) [file pone.0183446.s001.tif]

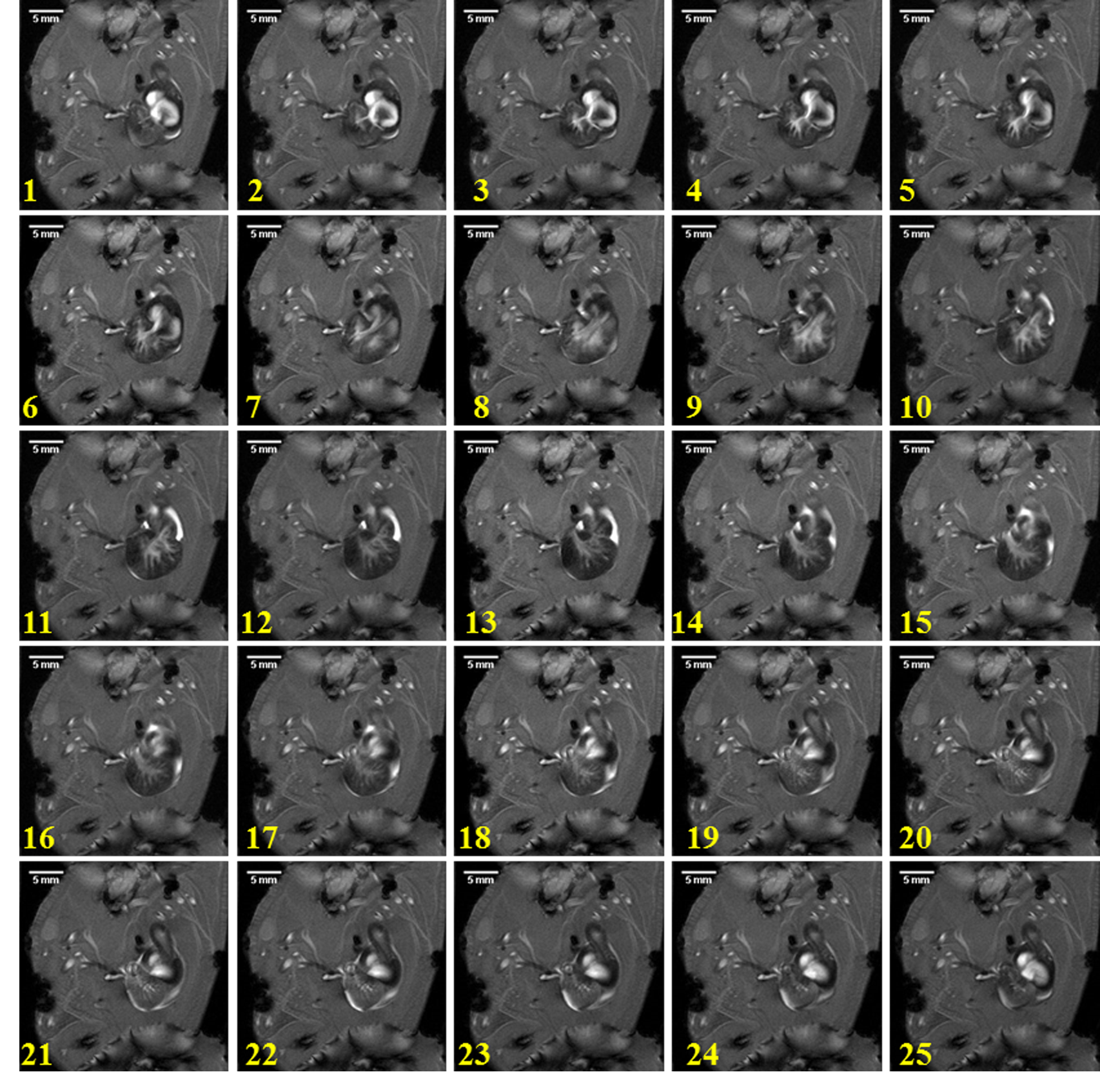

Supplement: S2 Fig — The heart is in systole in frame 1 and reaches diastole in frame 10. The order in which the atria, then ventricle and lastly the bulbus cordis & truncus arteriosus swell up with blood is very prominent when watching the frames as a short film (see S1 Video). (TIF) [file pone.0183446.s002.tif]

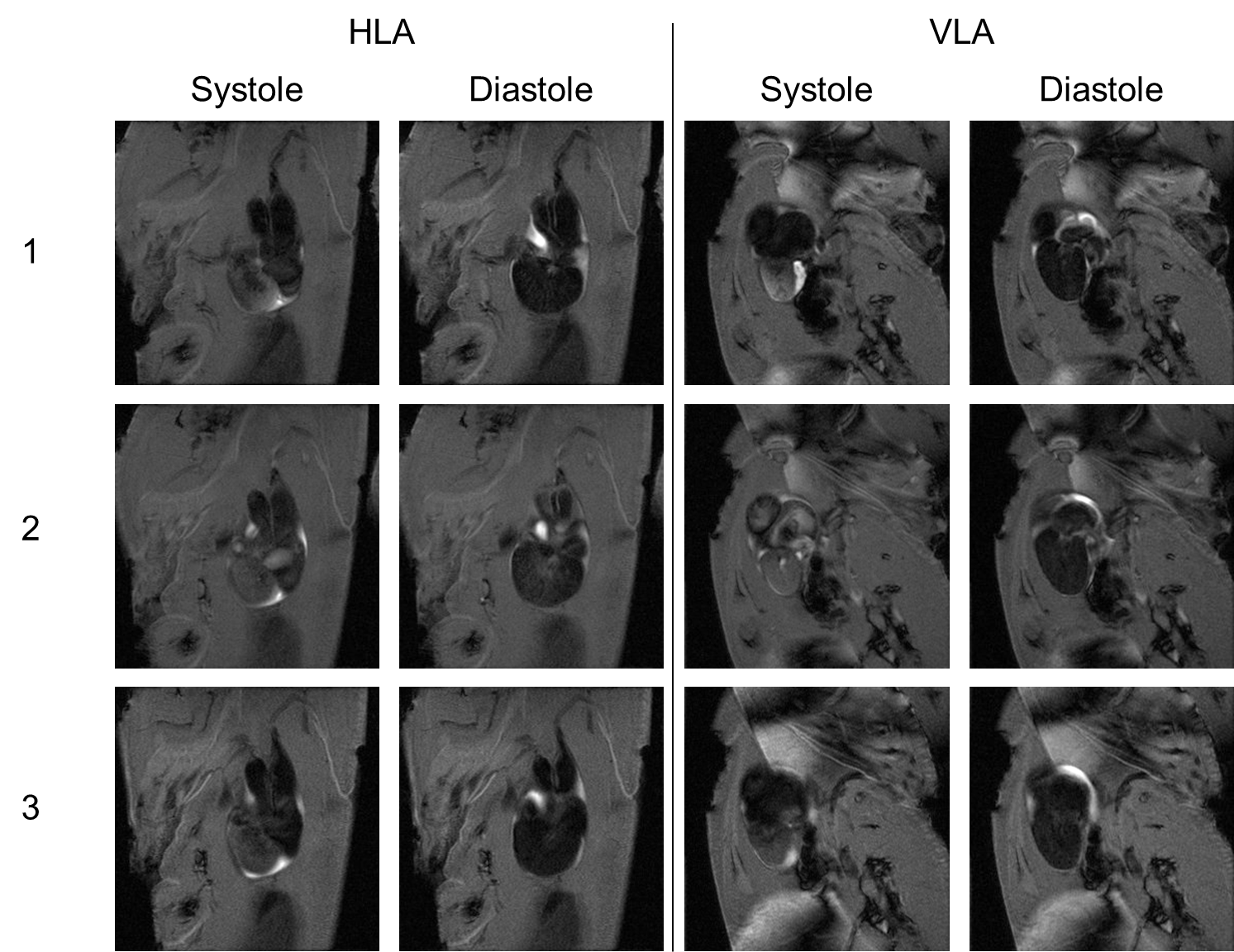

Supplement: S4 Fig — This image illustrates the consistency among repeated scans of the same animal. Mainly the size and position of the heart compartments are of importance when calculating the various cardiac function parameters. (TIF) [file pone.0183446.s004.tif]

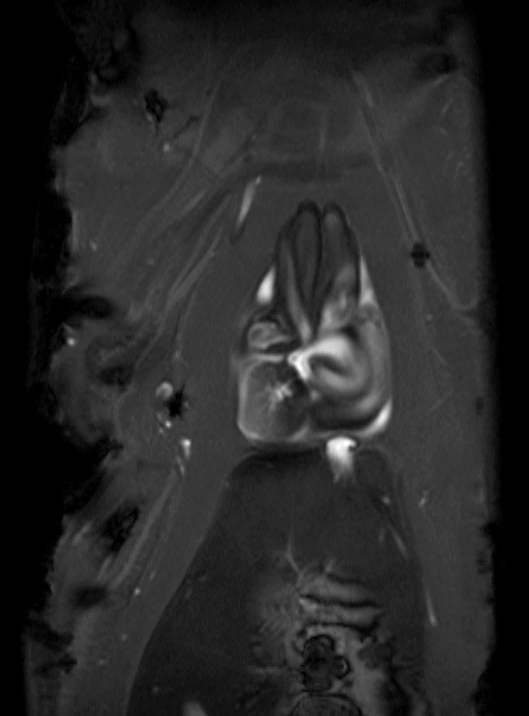

Supplement: S1 Video — This image was captured slightly off-plane from a coronal section of the body. The blood can be seen traveling from the atria to the single ventricle, before exiting the heart through the bulbus cordis. Motions seen at either the bottom or top of the screen result are the result of imaging artefacts. (GIF) [file pone.0183446.s007.gif]

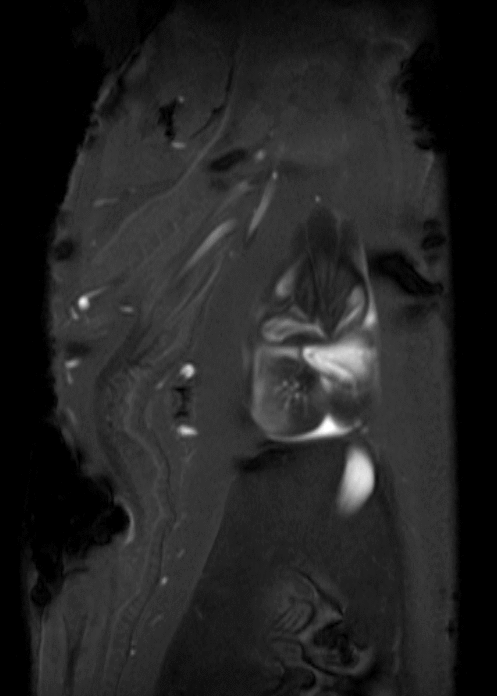

Supplement: S2 Video — This image is angled further from the coronal section of the body, and approaches the sagittal plane. The ventricle lies more predominantly in view, and the inner basket-weave structure of this compartment is highlighted. Motions seen at either the bottom or top of the screen are the result of imaging artefacts. (GIF) [file pone.0183446.s008.gif]

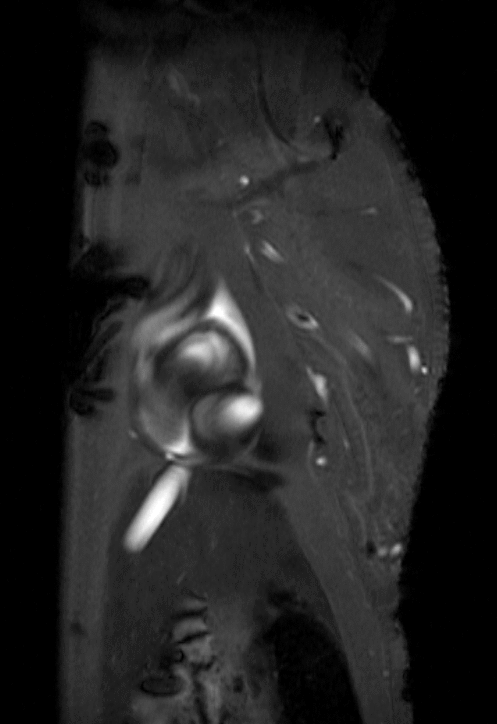

Supplement: S3 Video — This sagittal view reveals three of the compartments of the heart, namely the ventricle (lower left), one of the atria (top), and the sinus (lower right). Note how the atria fill up and blood is pushed into the ventricle. Simultaneously with the ventricle, the sinus will receive blood (coming back from the rest of the body). Motions seen at either the bottom or top of the screen are the result of imaging artefacts. (GIF) [file pone.0183446.s009.gif]
