# Supplementary material for: Novel axolotl cardiac function analysis method using magnetic resonance imaging
Source: PLoS One. 2017 Aug 24;12(8):e0183446. doi: 10.1371/journal.pone.0183446 (PMC5570274; doi:10.1371/journal.pone.0183446)
Supplement: S1 Table — (DOCX) [file pone.0183446.s005.docx]

| Measured by | HR (bpm) | EF (%) | SV (µL/beat) | CO (mL/min) |
| --- | --- | --- | --- | --- |
| LA | 32.17 ± 5.98 | 59.64 ± 10.8 | 133.72 ± 33.7 | 4.31 ± 1.09 |
| SA | 30.44 ± 5.51 | 48.07 ± 11.31 | 93.22 ± 31.19 | 2.84 ± 1.04 |
| US | 32.67 ± 4.92 | - | - | - |
