# Supplementary material for: Novel axolotl cardiac function analysis method using magnetic resonance imaging
Source: PLoS One. 2017 Aug 24;12(8):e0183446. doi: 10.1371/journal.pone.0183446 (PMC5570274; doi:10.1371/journal.pone.0183446)
Supplement: S2 Table — None of the cardiac function factors appeared to differ between the different animals and experimental repeats. This excludes experimental repeat of HR, in which the first experimental repeat appears to differ from the second and third. Using the LA or SA analysis techniques significantly affects the measurement of SV and EF, whereas HR was similar for LA, SA and US techniques. Bold = significant. (DOCX) [file pone.0183446.s006.docx]

| **Kruskal-Wallis** |  |  |  |  |  |
| --- | --- | --- | --- | --- | --- |
| Grouping variable: Repeat | |  |  |  |  |
|  | EF | SV | HR |  |  |
| Chi-Square | 2,169 | 2,211 | 17,378 |  |  |
| df | 2 | 2 | 2 |  |  |
| Asymp. Sig. | 0,338 | 0,331 | **0,000** |  |  |
|  |  |  |  |  |  |
| Grouping variable: Animals | |  |  |  |  |
|  | EF | SV | HR |  |  |
| Chi-Square | 4,042 | 3,52 | 0,309 |  |  |
| df | 2 | 2 | 2 |  |  |
| Asymp. Sig. | 0,133 | 0,172 | 0,857 |  |  |
|  |  |  |  |  |  |
| Grouping variable: Technique (LA and SA) | | | |  |  |
|  | EF | SV | HR |  |  |
| Chi-Square | 5,481 | 5,897 | 1,297 |  |  |
| df | 1 | 1 | 2 |  |  |
| Asymp. Sig. | **0,019** | **0,015** | 0,523 |  |  |
|  |  |  |  |  |  |
| **Wilcoxon test** |  |  |  |  |  |
|  | HRS - HRL | HRU - HRL | HRU - HRS | SVs - SVL | EFStest - EFLtest |
| Z | -,889^a^ | -,281^b^ | -1,838^b^ | -2,666^a^ | -2,429^a^ |
| Asymp. Sig. (2-tailed) | 0,374 | 0,779 | 0,066 | **0,008** | **0,015** |
|  | |  |  |  |  |
| a. Based on positive ranks. | |  |  |  |  |
| b. Based on negative ranks. | |  |  |  |  |
